# Doping Dependent Magnetic Behavior in MBE Grown GaAs_1-x_Sb_x_ Nanowires

**DOI:** 10.1038/s41598-020-65805-4

**Published:** 2020-06-02

**Authors:** Raj Kumar, Yang Liu, Jia Li, Shanthi Iyer, Lewis Reynolds

**Affiliations:** 1 Department of Materials Science and Engineering, North Carolina State University, Raleigh, NC 27695 USA; 2 Joint School of Nanoscience and Nanoengineering, North Carolina A&T State University, Greensboro, NC 27401 USA

**Keywords:** Materials science, Physics

## Abstract

Intrinsic and Te-doped GaAsSb nanowires with diameters ~100–120 nm were grown on a p-type Si(111) substrate by molecular beam epitaxy (MBE). Detailed magnetic, current/voltage and low-energy electron energy loss spectroscopy measurements were performed to investigate the effect of Te-doping. While intrinsic nanowires are diamagnetic over the temperature range 5–300 K, the Te-doped nanowires exhibit ferromagnetic behavior with the easy axis of magnetism perpendicular to the longitudinal axis of the nanowire. The temperature dependence of coercivity was analyzed and shown to be in agreement with a thermal activation model from 50–350 K but reveal more complex behavior in the low temperature regime. The EELS data show that Te doping introduced a high density of states (DOS) in the nanowire above the Fermi level in close proximity to the conduction band. The plausible origin of ferromagnetism in these Te-doped GaAsSb nanowires is discussed on the basis of d^0^ ferromagnetism, spin ordering of the Te dopants and the surface-state-induced magnetic ordering.

## Introduction

It has been nearly six decades since Wagner and Ellis^1^ reported the growth of Si whiskers via the vapor-liquid-solid (VLS) mechanism, which led to the extensive pursuit of research on semiconductor nanowires (see, for example, the perspective by Yang, *et al*.^2^ and the reviews in refs. ^3–5^). The driving force for such research growth has been the demonstration that reduced dimensions in our traditional electronic and optoelectronic devices lead to enhanced performance. The one-dimensional nature of nanowires (NWs) enables quantum confinement, strain relaxation in mismatched systems that is relevant for integration, band gap tuning and enhanced light trapping efficiency. Nanowires in both axial and core-shell heterostructure configurations have been grown and can give rise to unique electrical, optical and magnetic properties due to a high density of electronic states.

Furthermore, owing to their many potential applications in the emerging spintronic technologies, e.g., magnetic sensors, actuators and magnetic data storage, magnetic nanowires (NWs) and nanotubes have garnered much attention recently^6,7^. The key properties required for applications of magnetic NWs in spintronic devices include high saturation magnetization, control of the magnetization dynamics and a magnetic anisotropy energy along a preferred direction. Additionally, structure of magnetic nanowires could pay a way to realize the three-dimensional race-track type memory based on the domain wall motion technology^8^. Interestingly, high electron mobility and strong spin–orbit coupling of Indium-antimonide (InSb) and Indium-arsenide (InAs) nanowires could be harnessed for realizing Majorana-based topological quantum computers^9^. And as noted in our discussion below, there have been numerous investigations of magnetic behavior in metallic nanowires that suggest the easy axis of FM behavior is along the NW as opposed to that which we observe perpendicular to longitudinal axis.

Within the III-V materials, GaAsSb nanowires are particularly attractive due to their wide tunability of band gap within the 870–1700 nm (1.425–0.73 eV) regime of the electromagnetic spectrum and large carrier mobility (see ref. ^10^ and references therein). In addition, antimonide NW’s have a relatively large exciton Bohr radius below which one anticipates quantum confinement compared to other III-V semiconductors. In an axial configuration, these nanowires exhibit a pure zinc blende crystal structure with a reduced density of planar defects. The high structural and optical quality of GaAsSb axial nanowires has led to promising photodetectors^10,11^ and subwavelength wire lasers^12^ in the near-IR telecommunications wavelength range and high current density tunnel diodes^13^. Ganjipour, *et al*.^14^ fabricated a single hole transistor from a p-type GaSb nanowire and suggested that their results are relevant for spintronic applications and quantum information science since hole confinement in quantum systems can enhance the spin relaxation time. This is contrary to electron spins in quantum dots in which hyperfine interactions can lead to spin decoherence^15^. While there has been significant research on the electronic and optoelectronic properties of antimonide nanowires, there are limited reports on the magnetic behavior.

Both Sn-doped InSb and Te-doped GaSb whiskers with diameters in the 20–40 μm range exhibit negative magnetoresistance when the dopant concentration is near the metal-insulator transition^16,17^. Furthermore, Te-doped GaSb whiskers are reported to reveal a superconducting phase below 4 K^18^. Above the suggested superconducting transition temperature, resistivity of the whiskers shows metallic behavior. These authors attribute the magnetoresistance behavior with a weak anti-localization (WAL) model associated with sub-surface conducting channels. They also suggest that the large Rashba spin-orbit interaction parameter (~2–3 × 10^−12^ eV m) results in the observed magnetoresistance cusps at low fields in the longitudinal direction and their absence in the transverse direction. Additionally, Aharonov-Bohm oscillations and universal conductance fluctuations have been reported in GaAs/InAs core-shell nanowires^19^.

Here, we report dopant dependent ferromagnetism in GaAsSb nanowires with diameters ~100–120 nm grown by molecular beam epitaxy (MBE). In particular, while intrinsic nanowires are diamagnetic, Te-doped ones exhibit ferromagnetic behavior that is anisotropic. Potential mechanisms for ferromagnetism in these nonmagnetic atom doped nanowires are carrier-induced ferromagnetism (Zener’s mean field theory and double exchange), d^0^ ferromagnetism, spin ordering and surface-state-induced magnetic ordering. It is important to point out though that any carrier-mediated mechanism cannot be based on the typical sp-d hybridization since the d orbitals in Te are filled, suggesting that its magnetic moment is zero. The potential for spintronic applications of room temperature ferromagnetic (FM) semiconductors is significant. Suggested uses of such magnetic systems are magnetic filters, quantum transistors, nonvolatile magnetic memories, high density storage devices and spin qubits for quantum computing^20,21^. The FM behavior on the doped GaAsSb nanowires reported herein may provide a unique system in which to investigate spin transport, coherence and dynamics.

## Experimental

**Figure 1:**
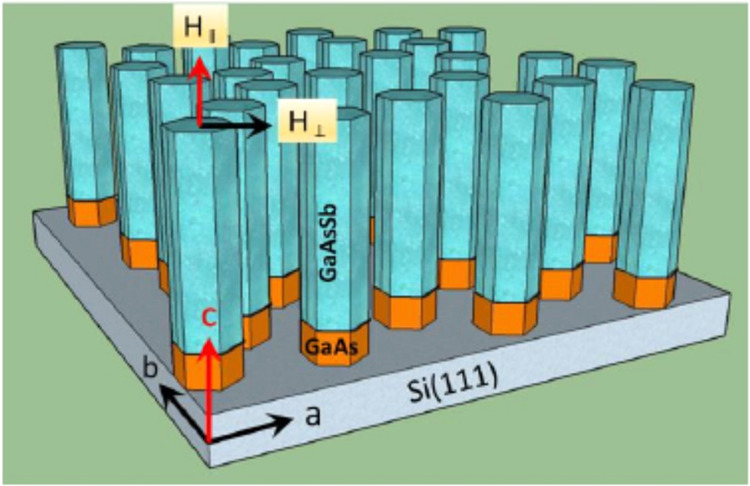
A schematic of Te-doped GaAs_1-x_Sb_x_ nanowires grown vertically on an p- Si(111) substrate showing magnetic field orientation with respect to the longitudinal axis of the nanowire.

The GaAs_1-x_Sb_x_ axial nanowires were grown vertically on a p-type Si (111) substrate by molecular beam epitaxy (MBE) as described in ref. ^22^. That is, an undoped GaAs stem is initially grown on the Si substrate followed by the GaAsSb segment (see Fig. 1). Scanning electron microscopy images of the intrinsic and Te- doped GaAsSb NWs are shown in Fig. 1(a,b) in the Supplementary Information file. High resolution Transmission electron microscopy (HRTEM) images and associated SAED patterns of top, middle and bottom of Te- doped GaAsSb NWs are shown in Fig. 2(i–n) in the Supplementary Information file. These images show that the nanowires have a zinc blende structure without any planar defects, twins or stacking faults. For these self-catalyzed nanowires, the As and Sb beam equivalent pressures were maintained at 4.8 × 10^−6^ and 4.8 × 10^−7^ torr, respectively, and growth occurred at 620 ^o^C. Energy dispersive X-ray spectroscopy in a scanning transmission electron microscope showed the Sb composition, x, to be ~7 at % for both the intrinsic and doped NWs. For the Te-doped nanowires investigated here, the electron carrier concentration for these growth conditions was previously^22^ estimated to be ~7 × 10^18^/cm^3^. We also grew nominally undoped GaAsSb nanowires as discussed below as a control for the magnetic measurements. The diameter of the Te-doped nanowires, ~120 nm, was ~20% greater than the intrinsic ones with a corresponding ~15% decrease in length with doping, which is consistent with the behavior reported by Suomalainen, *et al*.^23^ for the doping range of interest here. This behavior is associated with the surfactant effect of Te and its impact on the growth processes^22^.

The intrinsic GaAs_1-x_Sb_x_ nanowires were grown without any intentional dopant during MBE growth, and the Te-doped segment of the GaAsSb nanowires investigated here were grown on an intrinsic GaAs stem that would generate a pn junction. The total length of the NWs was ~5 µm. The current-voltage (I-V) characteristics were measured at room temperature on both the intrinsic and doped p-n nanowire arrays on the Si substrate to demonstrate the clearly different transport behavior between the two. The contact metals were Ti (50 nm)/Au (200 nm) and Ti (200 nm) on the top and back, respectively, as described by Kasanaboina, *et al*.^24^. The measured current in these NW arrays on the Si substrate is the sum of the currents through each individual nanowire using a two-probe technique. A detailed description of the fabrication sequence from our earlier work^22,24^ for the I-V measurements on these NW arrays is provided in the Supplementary Information.

Variable temperature magnetization measurements were conducted on 3 mm × 3 mm square samples in a Quantum Design® MPMS SQUID VSM magnetometer. The samples were mounted on a diamagnetic quartz sample holder using GE-7031 varnish and then loaded into the SQUID VSM using standard sample loading procedures. Magnetization measurements with two different orientations of the applied magnetic field relative to the longitudinal axis of the nanowires were performed to investigate anisotropy in the magnetic properties of these GaSb_x_As_1-x_ nanowires: 1) applied magnetic field (*H*_*||*_) was parallel to the longitudinal c- axis of the nanowires and 2) applied magnetic field (*H*_*⊥*_) was perpendicular to the longitudinal c-axis of nanowires. The magnetic measurements covered the temperature range from 5–350 K with magnetic fields 0–1 T.

The isothermal magnetic scans were performed as follows. The field was set to 0 by oscillating the 7 T magnet to zero the field and eliminate the presence of any remnant magnetic field in the superconducting magnet (see further comments in the supplementary information regarding the magnetic measurements). The sample was then cooled to 5 K in zero magnetic field, and the isothermal scans were performed at each temperature as the sample warmed from 5 to 350 K. To rule out any possibility of magnetic contamination during sample handling/loading, control experiments were performed on intrinsic GaSb_x_As_1−x_ nanowires samples using the same methods described above.

Typically, nominally undoped GaAsSb nanowires and thin films are p-type with a carrier concentration ~10^18^ cm^−3^. Prior investigations reported in the literature have ascribed this acceptor behavior to Ga antisite defects and/or Ga vacancies^25,26^. In our previous work on Te incorporation in GaAsSb nanowires, the Raman shift in the coupled plasmon-LO phonon mode from the uncoupled mode was used to estimate that the hole concentration in the intrinsic nanowires investigated here was ~2 × 10^18^ cm^−3^ ^22^.

## Results and Discussion

A schematic of vertically grown Te -doped GaAs_1-x_Sb_x_ nanowires on an p- Si(111) substrate is shown in Fig. 1. The magnetic field (*H*_*||*_) along the longitudinal c-axis of the NWs is shown by a red arrow while that (*H*_*⊥*_) in the radial (perpendicular) direction is denoted by a black arrow.

### I-V Measurements

**Figure 2:**
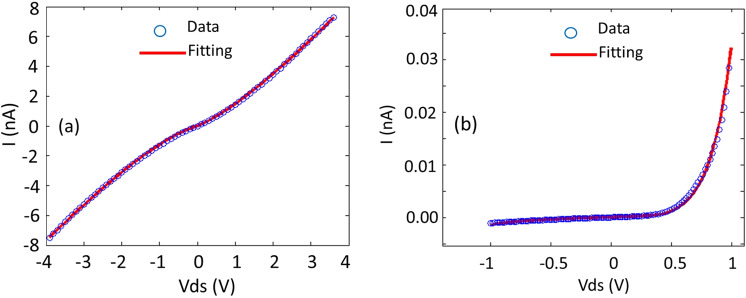
I-V measurements on (**a**) intrinsic and (**b**) Te-doped GaAs_1-x_Sb_x_ nanowires. The current is that for a single NW determined from the nanowire density and contact area.

I-V measurements on both the intrinsic and doped nanowire arrays are shown in Fig. 2(a,b), respectively. While the intrinsic NWs showed reasonably ohmic behavior as shown in Fig. 2(a), the Te-doped p-n ones revealed Schottky/diode behavior that was expected since the Te-doped segment of the nanowire was grown on an intrinsic segment as shown in Fig. 2(b). As mentioned above, intrinsic GaAsSb is typically p-type. Fitting of the I-V data using a Metal-Semiconductor-Metal (MSM) model^27,28^ resulted in symmetrical Schottky barrier heights (SBHs) of ~0.43 eV for both contacts and a hole concentration of ~10^18^ cm^−3^ for the intrinsic nanowire. This carrier concentration is consistent with that determined by the shift in the coupled plasmon-LO mode from the uncoupled mode in the Raman data.

For the Te-doped p-n nanowires, we obtained equivalent asymmetrical SBHs of ~0.8 and 1.0 eV that reflects the p-n nature of the doped nanowire and an equivalent electron concentration of ~5 × 10^18^ cm^−3^ for the doped one. COMSOL fitting has shown electron mobilities in the 1000–2000 cm^2^/Vs range. The difference in the Schottky barrier heights between the intrinsic and Te-doped NWs simply reflects that we have used a MSM model for a uniform nanowire, whereas the higher and asymmetrical barrier heights in the Te-doped case reflect the Schottky behavior associated with the doped GaAsSb segment. This differing behavior may indicate the presence of surface states that could modulate the magnetic behavior as discussed below. The combined effect of large surface to volume ratio and presence of surface states in nanowires may lead to modulation of the electronic, optical, and structural properties of nanowires, e.g., controlling the p-n junction switching behavior of GaAs nanowires via surface effects^29^. The pronounced effect of quantum confinement and reduced dimensionality on the magnetic properties of magnetic nanoparticles and thin films is well studied^21,30^. Due to reduced dimensionality in nanowires, the defect formation energy of surface states at the surface of nanowires is lower than the defect formation energy inside the nanowires. Moreover, the spin polarization energy of the surface states is higher than the defects in the bulk of nanowires which leads to a surface-state- induced strong magnetic ordering in magnetic nanowires^31^. Nevertheless, it is clear that the I-V characteristics in Fig. 2 exhibit different electrical transport behavior between intrinsic and doped nanowires. The question then is how this may relate to the magnetic data.

### Magnetometry measurments

**Figure 3:**
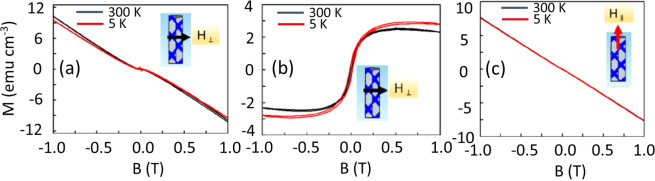
Isothermal M vs. H hysteresis plots of (**a**) intrinsic GaSb_x_As_1-x_ nanowires with a perpendicular magnetic field, (**b**) Te-doped GaSb_x_As_1-x_ nanowires with the same applied field perpendicular to the longitudinal axis of the nanowires and (**c**) Te-doped nanowires with the applied field parallel to the NW axis.

Figure 3 shows the Isothermal M vs. H measurements that were performed at 300 and 5 K on intrinsic and Te-doped GaAs_1-x_Sb_x_ nanowires with an applied perpendicular field orientation (*H*_*⊥*_). The intrinsic GaAs_1-x_Sb_x_ nanowires exhibit clear diamagnetic behavior at room temperature and 5 K while the Te-doped nanowires show strong ferromagnetic behavior with a saturation magnetization of ~3 emu/cm^3^, as shown in Fig. 3(a,b), respectively. The diamagnetic part of the sample and sample holder was subtracted from the data presented in Fig. 3(b). The diamagnetic behavior for the intrinsic nanowires rules out any possibility of magnetic contamination during sample preparation, handling and/or measurement in the SQUID VSM. In addition to the data in Fig. 3(a), isothermal M vs. H measurements were performed at the same temperatures on an intrinsic GaSb_x_As_1-x_ NW sample with an applied longitudinal field (*H*_*||*_), and the same diamagnetic behavior was observed. Figure 3(c) shows that the Te-doped nanowires are diamagnetic when the applied field is parallel to the longitudinal axis of the nanowires. That is, there is no magnetic response from the Te- doped GaSb_x_As_1-x_ nanowires when the applied magnetic field (*H*_*||*_) is applied along the longitudinal c-axis of the nanowires grown vertically on a p-Si(111) substrate. This is clear evidence of magnetic anisotropy in the doped nanowires with the easy axis of induced magnetism along the radial direction. This observation is contrary to the easy axis of magnetization parallel to the longitudinal axis in magnetic nanowires and consistent with an in-plane easy axis in thin films of ferromagnetic materials that results from the high demagnetization field along the surface normal to thin films. The demagnetization energy in magnetic thin films forces the magnetization to be parallel to the longest in-plane axis while in case of magnetic nanowires a stronger shape anisotropy pulls the magnetization out of plane and maintains the magnetization parallel to the longitudinal axis of FeCo metallic alloy nanowire arrays^32^. Having an in-plane magnetic anisotrpy in the Te- doped GaSb_x_As_1-x_ nanowires indicates that shape anisotropy is weak and other forms of anisotropy dominate to keep the magnetization easy axis along the radial direction of Te- doped GaSb_x_As_1-x_ nanowires. The fact that ferromagnetic behavior was only observed when the applied magnetic field was oriented perpendicular to the longitudinal axis of the nanowires and not along the NW confirms our earlier conclusion on the lack of any unintentional magnetic contamination associated with sample preparation and/or measurement.

**Figure 4:**
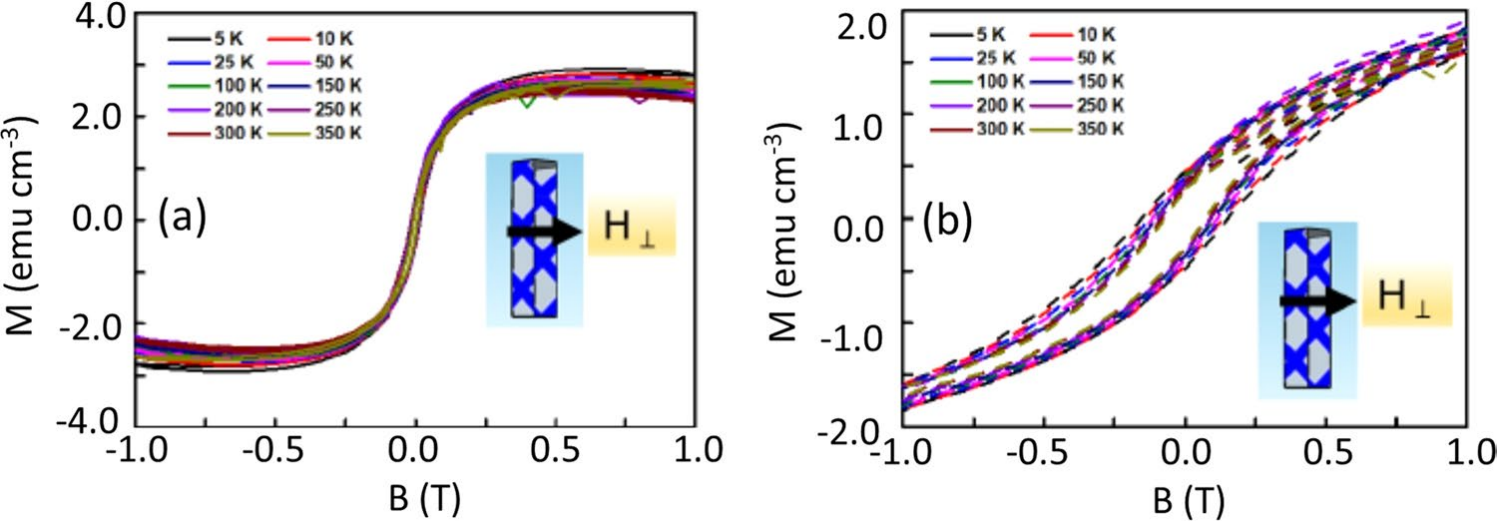
(**a**) M vs. H isothermal hysteresis plots of Te- doped GaSb_x_As_1-x_ nanowires with an applied magnetic field perpendicular to the longitudinal axis and (**b**) low field measurements in the same field orientation where the hysteresis behavior is clearly visible.

Isothermal M vs. H plots of Te- doped GaSb_x_As_1-x_ nanowires over the temperature range from 5–350 K with an applied perpendicular field (*H*_*⊥*_), i.e., field normal to the NW axis, are shown in Fig. 4. The diamagnetic part of the sample and sample holder was subtracted from the data presented in Fig. 4. As noted above, strong ferromagnetic behavior with a saturation magnetization of ~3 emu/cm^3^ was observed over the entire temperature range. The low field hysteresis plots in Fig. 4(b) reveal clear hysteresis behavior over the entire temperature range. The observed ferromagnetic beahvior was reproducible on multiple samples. The saturation in the magnetization and clear hysteresis loop at 350 K indicate that the Curie temperature is higher than 350 K for these Te- doped GaSb_x_As_1-x_ nanowires with a donor concentration of ~10^18^ cm^−3^, indicating robust ferromagnetic behavior at room temperature.

**Figure 5:**
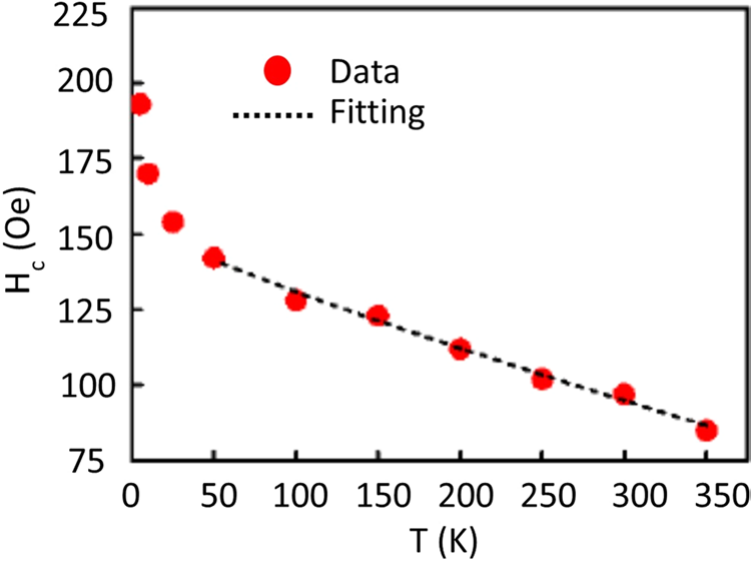
The magnetic coercivities of Te- doped GaSb_x_As_1-x_ nanowires as a function of temperature with an applied H_⊥_ magnetic field; the dashed line represents the fit for the dependence of the coercivity on temperature as shown in Eq. (1).

The coercivity (*H*_*c*_) values of our Te- doped GaSb_x_As_1-x_ nanowires at different temperatures were estimated from the isothermal M vs. H hysteresis plots shown in Fig. 4(b) and plotted as a function of temperature in Fig. 5. Two different regions of the temperature dependence of coercivity are clearly visible in Fig. 5. First, the corecivity monotonically increases from 350 K to 50 K below which there is an abrupt increase in slope of the coercivity vs temperature. The temperature dependence of coericivity in magnetic materials has been investigated for more than seven decades, and initial models from Néel ^33^ and Brown^34^ involved thermal activation over an energy barrier. More recent data^35,36^ on FM nanowires have shown that the coercivity decreases with increasing temperature. Our *H*_*c*_*(T)* data in Fig. 5 showing a more rapid decrease at lower temperatures are consistent with that reported by Zeng, *et al*.^36^ on Fe, Co and Ni nanowires. In addition to the tempertaure dependence of the intrinisic magnetic properties, thermal fluctuations can also play a crucial role in the temperature dependence of the coercivity in magnetic nanowires. Considering the shape anisotropy of the nanowires, the thermal activation-induced change in coercivity of nanowires can be explained by Eq. (1) where *H*_*c0*_, *M*_*s0*_, and *E*_00_ represent the coercivity, saturation magnetization and energy barrier at zero temperature, respectively. For an ideal case of aligned Stoner-Wohlfarth particles, m = 2, and for a nonsymmetric energy landscape as in case of nanowires^36^, the predicted value for the factor m = 3/2, which is consistent with Zeng’s data.

1 $${H}_{c}(T)={H}_{c0}\frac{{M}_{s}(T)}{{M}_{s0}}\lfloor 1-{\left(\frac{25{K}_{B}T{{M}_{s0}}^{2}}{{E}_{00}{M}_{s}^{2}(T)}\right)}^{1/m}\rfloor $$

Fitting the H_c_ vs T data from 350 K to 50 K using Eq. (1), the best fit as shown in Fig. 5 was obtained for m = 1.16, which indicates that the temperature dependence of the coercivity in these magnetic semiconductor nanowires cannot be solely explained by the thermal activation model which works well for traditional ferromagnetic materials. The temperautre dependence of coercivity in the low temperature regime of our magnetic nanowires is more complex as reported by Zeng *et al*.^36^. We were unable to fit the low tempearture coercivity data (25 K to 5 K) with Eq. (1), which is based on the thermal activation model of magnetization switching, and thus, further investigaton is needed to understand this anomaly in the low temperature coercivity behavior. Although Zeng, *et al*. mention the feasibility of m = 1, they provided no physical insight into its origin, again noting that the low temperature behavior is more complex than currently understood. Grobert^35^ suggests that a linear decrease of *H*_*c*_ vs *T* (m = 1) suggests the presence of anisotropy in their Fe-filled carbon nanotubes. However, Batlle, *et al*.^37^, suggested that m ~1.3 (corresponding to their k = 0.77) is consistent with an assembly of randomly oriented particles for their metal-doped BaFe_12_O_19_ compounds. This value of m is close to that we obtain for our fit to the *H*_*c*_ vs T data in Fig. 5. The main point here is that the temperature dependence of coercivity is not fully understood at present.

### Electron energy loss spectroscopy measurements(EELS)

**Figure 6:**
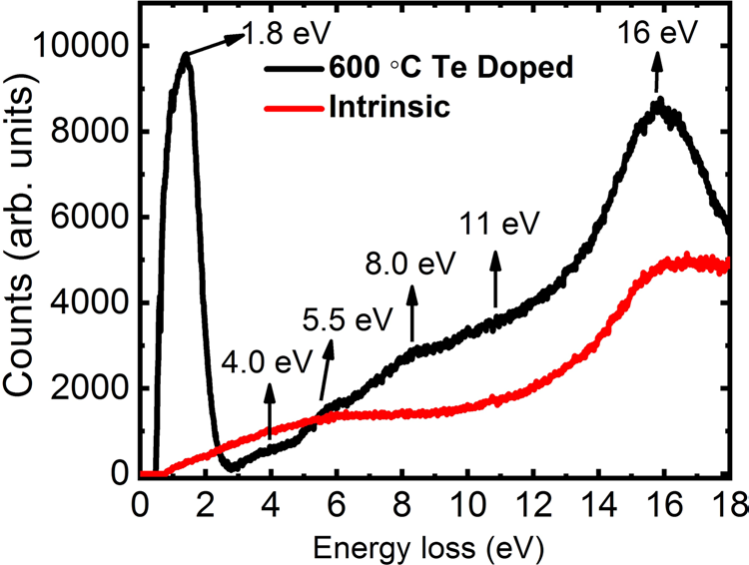
Electron energy loss spectra for intrinsic and Te-doped GaAs_1-x_Sb_x_ nanowires.

Monochromated electron energy loss spectroscopic (EELS) data were generated on intrinsic and Te-doped GaAsSb nanowires to potentially achieve insight on the origin of the ferromagnetic behavior observed in the doped nanowires in the in-plane configuration. As mentioned below, the zero loss peak of the EEL spectra was subtracted, and the resultant subtracted EELS results for the intrinsic and Te-doped nanowires are shown in Fig. 6. In this regime, the EELS spectra are dominated by excitation of interband transitions and plasmons, which are collective oscillations of valence electrons.

As shown in Fig. 6, the EEL spectrum of the low loss region (0 to 18 eV) exhibits significant changes after Te doping. Several peaks at energy loss of 1.8 eV, 4 eV, 5.5 eV, 8 eV, 11 eV and 16 eV are present in the low loss region in the EELS for the Te-doped nanowires whereas that of the intrinsic nanowires only show two board peaks at ~5.5 eV and 16 eV. Since the peaks at 5.5 and 11 eV, and also those at 4, 8 and 16 eV, are multiples of each other, one can infer that they involve energy doubling from the plasma peak. The valence electron plasma energy has been reported to be 15.95 and 13.98 eV for GaAs and GaSb^38^, respectively, which is near the 16 eV peak in Fig. 6. Generally, EELS probes empty states above the Fermi level. The electrons that are excited from the valence band by the incident electrons can only scatter into available unoccupied states above the Fermi level. Particularly, the first peak at 1.8 eV in the Te-doped nanowire is significant, indicating that the Te doping has introduced a high density of states (DOS) in the nanowire above the Fermi level in close proximity to the conduction band. However, one must note that this energy is greater than the band gap (~1.3 eV), suggesting that it involves an interband transition from a state deeper in the valence band. It may also represent a complex involving Te or the presence of surface states. In some cases, phenomena from surface states and from states in the band gap are indistinguishable in EELS. Based on our current experimental data, we are unable to identify the origin of the 1.8 eV peak in the doped NWs. Nevertheless, the presence of the 1.8 eV peak in the EELS spectra of the Te-doped nanowires but not in the intrinsic ones strongly suggests that doping creates levels in the band structure, which most likely provides some insight on our observation of ferromagnetic behavior and the associated anisotropic magnetic properties of the doped nanowires compared to their lack in the intrinsic ones.

There are several conclusions with regard to the observation of ferromagnetic behavior in our Te-doped GaAsSb nanowires. Anisotropy of the magnetic behavior with the easy axis perpendicular to the axis of the nanowire and diamagnetism when the field is applied along the NW axis implies that the existence of FM is intrinsic. This is in contrast to metallic ferromagnetic nanowires, in which the easy axis is along the NW axis^32,36^. In these metallic nanowires the magnetic anisotropy was dominated by shape anisotropy. Furthermore, the fact that ferromagnetism is observed in our doped semiconductor nanowires but not intrinsic ones suggests that it is dopant induced. We note that the doping concentration in our doped nanowires, mid-10^18^ cm^−3^, is in the vicinity of the metal-insulator transition (MIT) as reported by Druzhinin, *et al*.^17,18^, which was relevant to their magnetoresistance (MR) behavior. They demonstrated that negative MR due to strong magnetic ordering occurred only when the carrier concentration of the non-magnetic Te impurities was within the MIT regime. The issue then is what is the origin of the long range magnetic ordering in our doped nanowires. While one is tempted to attribute the ferromagnetism to a carrier-mediated mechanism via an exchange interaction as discussed by Dietl, *et al*.^39^, there is no magnetic species in our nanowires. In fact, the Curie temperature *T*_*c*_ predicted for GaSb containing 5% Mn and 3.5 × 10^20^ holes cm^−3^ is ~40 K^39^, which is substantially below our observed FM at room temperature. The expected *T*_*c*_ would be even lower for lower hole concentrations if one considers the doping dependence of *T*_*c*_ for (Ga,Mn)Sb reported by Ohno^40^, in which *T*_*c*_ is proportional to p^0.78^. This does not exclude a Fermi level effect since our EELS data imply a large density of states above the Fermi level in the Te-doped nanowires compared to the intrinsic ones, but any mechanism must differ from that discussed by Dietl^39^, El-Masry^20,41^ and Arkun^42^, again since there is no magnetic species in our nanowires. One should also note that the diameter of these nanowires, ~100–120 nm, is significantly greater than the Bohr radius for our 7 at % Sb composition, suggesting that quantum confinement should not be a factor in our observed FM behavior.

The origin of ferromagnetism is typically associated with partially filled 3d or 4 f orbitals. However, as noted earlier, since the 4d-orbitals for Te in our doped nanowires are filled and thus have no unpaired spins, one cannot associate our FM behavior with unpaired spins in these orbitals nor can we invoke sp-d hybridization that has been used in previous carrier-induced mechanisms. However, there are unpaired spins in the 5p orbital for Te, but these electrons tend to be less localized than inner shell d-electrons. More recently, others^42–45^ have attributed ferromagnetism in materials without magnetic ions to lattice or bond defects and referred to this as d^0^ ferromagnetism. Schoenhalz, *et al*.^46^ used total energy calculations to demonstrate that the long-range magnetic interactions in undoped ZnO nanostructures are related to extended defects such as surfaces whereas Mal, *et al*.^47^ have attributed FM in undoped ZnO to oxygen vacancies. Comparison of the magnetic data in Fig. 3 between intrinsic and doped nanowires suggests that Te doping creates delocalized spin states. More recently, Ruiz, *et al*. performed magnetic measurements on single FeSi nanowires and associate the presence of ferromagnetism to an interaction between charge carriers and dangling bonds on the NW surface^48^. One of the most relevant investigations to our doping dependent FM in GaAsSb nanowires is that reported by Liu, et al.^49^ in which they explore the interaction between magnetic moments and itinerate carriers in d^0^ 4H-SiC single crystals that are ferromagnetic. They used Al and N ion implantation into the semi-insulating SiC to generate p- and n-type free carriers and defects and suggest that the magnetic moments are related to the C p orbitals. The implanted wafer was diamagnetic prior to pulsed laser annealing but exhibited increasing FM order with increasing laser energy density up to 0.6 J cm^−2^. For a wafer annealed at 0.6 J cm^−2^, they further demonstrated diamagnetic behavior when the applied field was oriented perpendicular to the wafer (000–1) surface but FM in the parallel direction. Based on their data then, they suggest that p-type doping can enhance defect-induced FM while n-type doping suppresses it although others^50,51^ have shown that negatively charged (V_Si_V_C_) divacancies induce FM spin ordering. In contrast, our high-resolution scanning transmission electron micrographs show a pure ZB crystal structure without planar defects or twins. However, we note that the p-type behavior of the underlying GaAs stem results from Ga vacancies as pointed out earlier. Another potential origin of ferromagnetism in our doped nanowires may lie in strong magnetic ordering of the 5p spins on the Te dopant atoms when the dopant concentration is near the MIT as suggested by Druzhinin, *et al*.^17,18^ and is the case in our samples. Coey^52^ has recently discussed the complexity of temperature independent d^0^ FM in oxides with non-magnetic cations and concluded that currently available data cannot be explained via our conventional understanding. He points out that surface defects are responsible and has suggested two potential hypotheses, a spin-split impurity band or giant orbital paramagnetism. Furthermore, a different I-V behavior observed in our intrinsic and Te- doped GaAsSb nanowires (see Fig. 2) may indicate the presence of surface states. The surface states induced enhancement in the spin-polarization energy which leads to magnetic ordering also offers another plausible explanation for the observed magnetic ordering in Te-doped GaAsSb nanowires^29–31^. Further investigation is required to elucidate the mechanism for our dopant-induced magnetic behavior more definitively.

## Conclusion

In conclusion, the magnetic properties of MBE grown Te -doped GaAsSb ferromagnetic nanowire arrays have been investigated over the temperature range from 5–300 K. Our data reveal ferromagnetic behavior when the applied field is oriented perpendicular to the longitudinal axis of the nanowires for doped nanowires but diamagnetic for intrinsic GaAsSb. The EELS spectral data on the Te-doped nanowires reveal the presence of a high density of states above the Fermi level in close proximity to the conduction band. The temperature dependence of the coercivity agrees with a thermal activation model with an estimated value of m = 1.16 in the exponent of Eq. (1) over the temperature range from 50–300 K but shows a more complex behavior at lower temperatures. The origin of ferromagnetism in our doped GaAsSb nanowires is discussed in terms of d^0^ ferromagnetism and spin ordering of the Te dopants. Further experimental investigation and theoretical modelling are required to understand the magnetization dynamics in the low temperature regime and provide a conclusive origin for the observation of ferromagnetism in these Te- doped GaAsSb nanowires. But, our data clearly show that the statement^49^ materials with s or p electrons are only ferromagnetic when structural/point defects, for example, vacancies, are present may not necessarily be true since Te is an intentionally added dopant impurity here.

## Supplementary information

Supplementary information.

## Data Availability

All data generated or analyzed during this study are included in this published article (and its supplementary information file).
