# Supplementary material for: Doping Dependent Magnetic Behavior in MBE Grown GaAs1-xSbx Nanowires
Source: Sci Rep. 2020 Jun 2;10:8995. doi: 10.1038/s41598-020-65805-4 (PMC7265495; doi:10.1038/s41598-020-65805-4)
Supplement: Supplementary file 1 — Supplementary information. [file 41598_2020_65805_MOESM1_ESM.pdf]

Supplementary Information

Doping Dependent Magnetic Behavior in MBE Grown $GaAs_{1-x}Sb_x$ Nanowires

Raj Kumar,[1] Yang Liu,[1] Jia Li,[2] Shanthi Iyer[2] and Lewis Reynolds Jr.[1]

[1]Department of Materials Science and Engineering, North Carolina State University, Raleigh, NC 27695

[2]Joint School of Nanoscience and Nanoengineering, North Carolina A&T State University, Greensboro, NC 27401

(1) Scanning electron microscope images of intrinsic and doped GaAsSb nanowires from Ahmad, et al, Semicond. Sci. Technol. **31**, 125001(2016) are shown below. The data show that the axial and radial growth rates are affected by Te-doping; that is, the axial growth rate decreases with increased Te dopant whereas the radial growth rate increases. One should also note that the nanowires are tapered. These trends are consistent with that reported by Suomakainen, et al, Appl. Phys. Lett. **107**, 012101(2015) as noted in the manuscript.

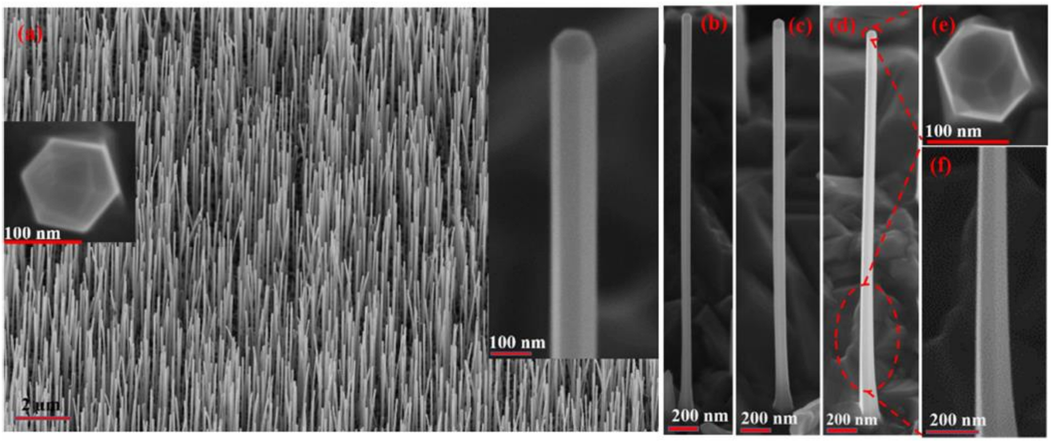

Fig. 1. (a) SEM image of a 45° tilted Te (500) -doped nanowire, insets show the well defiend hexagonal facets of a single magnified NW; (b), (c) and (d) show the intrinsic, Te (600) -doped nanowires; (e), (f) display magnification images of the top and lower sections of a Te (600) -doped NW.

(2) High-resolution transmission electron micrographs and the associated selected area electron diffraction patterns near the top, middle and bottom of a nanowire that was Te-doped (600°C Te effusion cell temperature) are shown in Fig. 2 (i, j, k, l, m, n) (from Ahmad, et al, Semicond. Sci. Technol.**31**, 125001(2016)). The SAED patterns reveal that the crystal structure is zinc blende with no evidence of wurtzite; the lack of satellite spots implies that no twins are present. The HRTEM images reveal no evidence of planar defects. Both observations suggest the high quality of these GaAsSb NWs.

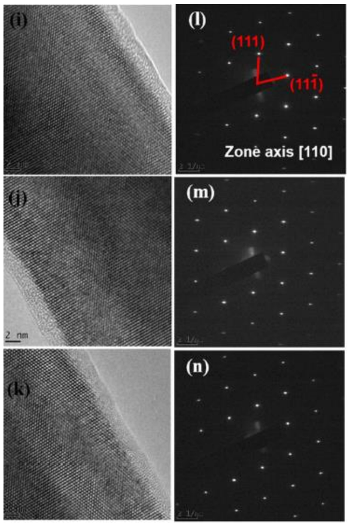

Fig. 2. (i) HRTEM images of the top; (j) middle and, (k) bottom of Te-doped GaAsSb NWs; (l) SAED patterns of top; (m) SAED patterns of middle; (n) SAED patterns of bottom of Te-doped GaAsSb NWs; Note the lack of satellite peaks in the latter.

(3) Fabrication sequence for contacts on GaAsSb NWs for performing I-V measurements on arrays. Since the measurements were performed on arrays, the total current is the sum of currents through each nanowire to which contact is made using a two-probe technique with a Keithley 4200. First, PMMA was spun onto an array of nanowires on the Si substrate and then plasma etched to expose NW tips.

The top contact on the NWs was an array of 1 mm diameter circular contact pads with the metal being 50 nm Ti/200 nm Au. The back metal e-beam deposited on the bottom of the substrate was 200 nm Au. This process was originally described in Kasanaboina, et al, Nanoscale Res. Lett. **11**:47(2016).
